# Paradoxical leadership and well-being in turbulent times: a time-lagged study

**DOI:** 10.3389/fpsyg.2023.1148822

**Published:** 2023-11-24

**Authors:** Dave Stynen, Judith Semeijn

**Affiliations:** ^1^Department of Organization, Faculty of Management, Open Universiteit, Heerlen, Netherlands; ^2^Department of Epidemiology, Faculty of Health, Medicine and Life Sciences, Maastricht University, Maastricht, Netherlands; ^3^Research Centre for Education and the Labour Market, School of Business and Economics, Maastricht University, Maastricht, Netherlands

**Keywords:** career satisfaction, life satisfaction, job insecurity, paradoxical leadership, sense-making theory, well-being

## Abstract

**Introduction:**

Paradoxical leadership has recently been put forward as an approach to leadership that may transcend the inherent contradictions in contemporary organizational and personnel management. Empirical research on its potential role for bolstering employee well-being remains scarce. This study investigated whether paradoxical leadership positively impacts employee well-being, which is operationalized as employees’ job, career and life satisfaction. We rely on sense-making theory to investigate whether such effects are mediated by the mitigation of employee job insecurity perceptions.

**Methods:**

Convenience sampling techniques were used to collect longitudinal survey data between March and September 2021. In total 287 workers provided usable data. Their ages ranged from 18 to 67 years and were active in various organizations in the Netherlands. Validated measures were used to assess paradoxical leadership, job insecurity, job, career, and life satisfaction. A time-lagged path analysis in Mplus 7.0 was conducted to investigate relationships.

**Results:**

The results suggest that paradoxical leadership is positively related to job, career and life satisfaction over time. The relationships between paradoxical leadership and job and career satisfaction are partially mediated by the mitigation of perceived job insecurity.

**Discussion:**

Paradoxical leadership plays a role in fostering worker well-being in these turbulent times. Paradoxical leaders may also help their followers to reframe and better deal with challenging working conditions. Despite the longitudinal data design, an additional data-wave would allow for more stringent testing of the proposed mediation effect, and due to convenience sampling generalization of findings is limited.

## 1 Introduction

In a recent report of the World Health Organization [WHO] and International Labour Organization [ILO] (2022) work is more than ever regarded as both an opportunity and a risk for worker health and well-being. The risks have been amplified over the last decade as macro-economic developments, like technological disruptions, population aging, and the globalization of production and service chains led to important changes in the working environment (e.g., introduction of artificial intelligence, restructuring, flexibilization of labor) and thereby to increased pressure and stress and uncertainty as well (Kalleberg, 2011; Di Fabio, 2017; Engbersen et al., 2020; Yam et al., 2023). More recently, global crises like COVID-19 further impacted employment and career prospects in various sectors as well as the well-being of workers and their families in a negative way (Handwerker et al., 2020; Gaspar et al., 2021). Hence, more action is needed to foster worker well-being as their well-being is also intertwined with the well-being of their communities, and efforts could therefore aid the realization of SDG-3 (Good health and well-being) (International Labour Organization [ILO], 2022; World Health Organization [WHO] and International Labour Organization [ILO], 2022; Nilsen and Kongsvik, 2023).

Fostering employee well-being is hence an increasing concern in today’s organizational practice (Huettermann and Bruch, 2019). A key facet of well-being at work is job satisfaction, or the positive emotional state resulting from an employees’ job appraisal and may encompass facets like job conditions, job content and social relationships (Locke, 1976; Spector, 1997). Abundant research has investigated how organizations can provide work opportunities that facilitate employee well-being through human resource management (HRM) and leadership practices (Den Hartog et al., 2013; Inceoglu et al., 2018; Das and Pattanayak, 2023). However, these insights (and related practices) may not fully address the inherent *paradoxical tensions* (e.g., efficiency vs. innovation, long term vs. short term, task vs. people oriented) that characterizes these turbulent times and therefore run the risk of falling short. One of the key concerns to date for personnel management is how to reconcile, in HRM practices and leadership, the foci on employee well-being and organizational performance (Salas-Vallina et al., 2021).

The role of leaders is considered vital in supporting workers exposed to challenging and stressful work environments in order to maintain employee well-being (Harms et al., 2017; Inceoglu et al., 2018). In the leadership literature *paradoxical leadership* has recently been put forward as an approach that may transcend the inherent contradictions in contemporary organizational and personnel management (Zhang et al., 2015, 2021, 2022). It is grounded in Eastern philosophy on handling paradoxes, which can embrace, integrate and transcend opposites. Western approaches to handling of paradoxes is merely analytical and considers opposites as separate parts, instead of considering them as a whole. According to Zhang et al. (2015) paradoxical leadership invites us to capture paradoxes from an Eastern perspective that encompasses a set of behaviors that may appear to be contradictory, yet interrelated. These can, when applied over a longer time window, meet the competing demands of modern workplaces. Paradoxical leadership has been linked in a positive way to several different facets of favorable employee behavior including (innovative) employee performance (Li et al., 2018), voice behavior (Li et al., 2020; Xue et al., 2020; Shehata et al., 2023), creativity (Zhang et al., 2022) and organizational citizenship behavior (Li et al., 2018, 2020; Xue et al., 2020; Meng et al., 2021; Zhang et al., 2021; Liu and Pak, 2023; Shehata et al., 2023). In contrast to employee behavior as an outcome, fewer studies have looked at employee well-being. Recent studies have uncovered positive linkages with work engagement (Fürstenberg et al., 2021; Shehata et al., 2023) and psychological well-being (Li et al., 2022). In particular the relationship between paradoxical leadership and job satisfaction has barely been studied empirically. Only one study reported a positive relationship in the public sector (Backhaus et al., 2022). This study’s objective is first of all to further close this gap by empirically exploring the relationship between paradoxical leadership and an overlooked aspect of employee well-being, i.e., job satisfaction. In addition, we want to extend research on paradoxical leadership and employee wellbeing as an outcome by investigating to what extent paradoxical leadership may also spill over to well-being beyond one’s current job, in terms of career and life satisfaction. These days work and non-work domains are permeable and careers more boundaryless (Guan et al., 2019; McDaniel et al., 2021). Therefore, career satisfaction – or the accumulated evaluation of one’s career so far- and life satisfaction, or the overall appraisal of one’s life (Diener et al., 1985; Hagmaier et al., 2018) are studied as outcomes in addition to job satisfaction. Thus far, these relationships have not been studied empirically. Hence, our second research objective is to extend research on paradoxical leadership to well-being beyond one’s job.

The third objective of this study is to enhance our understanding of the explanatory mechanisms that underly the relationship between paradoxical leadership and well-being. This has been called upon by scholars like Fürstenberg et al. (2021). We build and extend Fürstenberg et al. (2021) and Zhang et al. (2021)’s recent theorizing regarding the effects of paradoxical leadership by relying on sense-making theory (Weick, 1995) and the Job Demands-Resources Model (JD-R, Demerouti et al., 2001). It has been proposed that paradoxical leaders, through consciously combining opposing behaviors, may nurture the experienced resourcefulness of the working environment, like enhancing employee autonomy and goal clarity (Fürstenberg et al., 2021). The way paradoxical leaders can alter the working environment is through stimulating sense-making among followers. In the face of an increasingly challenging working environment, paradoxical leaders can sense and shape both the productive opportunities or job resources and redefine the threats or job demands in a more malleable way (Zhang et al., 2021). Thus far others have empirically demonstrated that paradoxical leadership positively contributes to the experienced job resources. We propose that paradoxical leaders may also mitigate experienced job demands. The underlying tenet is that through their behavior leaders may shape followers’ demanding job conditions, as established in earlier research (e.g., Tuckey et al., 2012). The job demand we put forward in this study is job insecurity, or the perceived threats to the job itself (Greenhalgh and Rosenblatt, 1984). Job insecurity is an established work stressor (Langerak et al., 2022) with negative consequences for employee satisfaction which includes job satisfaction, career satisfaction and life satisfaction (Otto et al., 2011; Alarco et al., 2012; Jiang and Lavaysse, 2018). Research has indicated that this stressor is linked to economic turmoil in the labor market (e.g., unemployment rates) as well as organizational changes (e.g., downsizing) (Shoss, 2017). Uncertainty regarding one’s role in the organization is inherent to paradoxes (Maitlis and Sonenshein, 2010) and triggers sense-making processes in workers (Zhang et al., 2021). Hence, this study investigates whether paradoxical leadership may mitigate perceptions of job insecurity, and thereby can protect employee well-being.

In sum, this study contributes in several ways to the literature. First, it investigates how a timely leadership style like paradoxical leadership may contribute to employee job satisfaction (Backhaus et al., 2022) and spill over to well-being beyond one’s job (career satisfaction and life satisfaction). Second, by investigating the mediating role of job insecurity, we contribute to theorizing on the effects of leadership styles on employee well-being (Inceoglu et al., 2018). Specifically, we elaborate on how leaders may shape their followers’ perceived job demands by making use of sense-making theory (Weick, 1995) in addition to the Job Demands-Resources Model (JD-R, Demerouti et al., 2001). Finally, we also add to the literature on the determinants of job insecurity, wherein the role of leadership as an interpersonal factor has barely been studied in comparison to personal, macro-economic and organizational factors (Shoss, 2017). These relations are studied by means of an online time-lagged survey among Dutch employees.

## 2 Theory and hypotheses

### 2.1 Paradoxical leadership

Leadership has been defined in several ways. Common ground can be found regarding “*an emphasis on the social influence process it involves, whereby leaders facilitate individual and collective efforts to accomplish common goals*” (Oreg and Berson, 2019, p. 273). Typically, leadership literature has mainly been concerned with employee and organizational performance (Inceoglu et al., 2018), with some portraying it as one of the most important contributing factors (e.g., Zaccaro et al., 2001). What constitutes leadership has been sought in both leader’s personal characteristics (e.g., personality, demographic variables) and leadership behaviors, which are more stable leadership styles that exceed specific situations (Oreg and Berson, 2019). Over the last decades several leadership styles have been identified ranging from more task-oriented (e.g., transactional leadership), relational-oriented (e.g., participative leadership, servant leadership), change-oriented (e.g., charismatic and transformational leadership) and passive leadership (e.g., laissez-faire leadership) (Derue et al., 2011; Yukl, 2012; Inceoglu et al., 2018).

Paradoxical leadership is a constructive leadership style that transcends these seemingly contradictory aforementioned behavioral orientations and fits with managing the paradoxical issues that organizations are facing in globalized markets where digitization and innovative technology are developing at a rapid pace (Zhang et al., 2015). A paradoxical organizational problem consists of contradictory but interrelated elements that are presented simultaneously and for a long time (Smith and Lewis, 2011). They operate between opposing elements that “*seem logical individually but inconsistent and even absurd when juxtaposed*” (Smith and Lewis, 2011, p. 382). Yet, these opposing elements are highly interdependent, because they are bounded simultaneously to the opposing poles (Smith and Lewis, 2011). Therefore, separation is unfruitful, and the opposing elements in paradoxical tensions should be addressed simultaneously to sustain organizational performance over the long term (Lewis, 2000; Smith and Lewis, 2011; Hahn and Knight, 2021). Prototypical examples are the tension between profitability and responsibility, long term versus short term, control versus freedom, differentiation versus integration (Clegg et al., 2002; Bloodgood and Chae, 2010). Also, the day-to-day people management is beset with paradoxical tensions and paradoxical leadership, which encompasses a set of behaviors that may appear to be contradictory, and is likely to attune to the competing demands of modern workplaces (Zhang et al., 2015).

The behavioral style of a paradoxical leader can be situated on five dimensions that consists of two “sides” that depend on and complement each other (Zhang et al., 2015). In situational and contingency perspectives on leadership, effective leadership behaviors is a matter of choosing for being for instance directive or participative (“either/or”) depending on the demands of the work context. In contrast, paradoxical leadership implies behaviors that one is accepting and aims to harmonize or integrate (“both/and”) competing demands (Zhang et al., 2015). Effective long term paradoxical leaders are assumed to (1) combine self-centeredness with other centeredness, (2) maintain both distance and closeness, (3) treat followers uniformly while allowing for individualization, (4) enforce work requirements while allowing flexibility and (5) maintain decision control while allowing autonomy. The first dimension refers to the ability of a leader to remain the central influential source on the work floor while also tuning in to workers’ needs and allow for shared leadership with followers. The second dimension comprises of the ability to maintain hierarchical differences in resolving work-related issues, but at the same time build strong interpersonal relationships with followers. The third dimension is about ensuring harmony between uniformity and individuality by treating employees equally based on agreements and rules, while simultaneously making distinctions based on individual’s wishes and talents. Finally, the last two dimensions both relate to the tension between control and empowerment. While the fourth concerns the ability to exert behavioral control (e.g., work processes) while allowing for flexibility, the fifth refers to the ability to maintain decision control (as in output control) while simultaneously stimulating employee autonomy.

Empirical studies have demonstrated that managers who exhibit paradoxical leadership are most effective in dealing with conflicting organizational issues in the short and long term (Pearce et al., 2019). Also, the beneficial consequences for organizational and employee performance have been demonstrated which includes indicators such as task performance (Zhang et al., 2015, 2021) as well as indicators of contextual performance, including creativity (Shao et al., 2019; Yang et al., 2021), innovative behaviors (Ingram et al., 2016; Zhang et al., 2021), adaptability (Zhang et al., 2021), voice behavior, and organizational citizenship behavior (Li et al., 2018, 2020; Xue et al., 2020; Meng et al., 2021; Zhang et al., 2021; Liu and Pak, 2023; Shehata et al., 2023).

As with most leadership styles, implications for employee well-being remains far less studied compared to performance outcomes (Inceoglu et al., 2018). In this study we look into employee well-being by examining three concepts that serve as our outcome variables: job satisfaction, career satisfaction and life satisfaction.

### 2.2 Indicators of well-being: job, career and life satisfaction

Job satisfaction refers to the subjective well-being of individuals at work (Judge et al., 2020) and builds on Locke’s (1976) original definition, which is “*a pleasurable or positive emotional state resulting from the appraisal of one’s job or job experiences*” (p. 1304). In this study, we focus on global job satisfaction or the overall affect that workers experience regarding their job, and not on the satisfaction with particular job features (e.g., pay, co-workers) (Bowling and Hammond, 2008). Over time, job satisfaction has been studied extensively and appeared important for various other and more distal outcomes including individual motivation and performance, which is also of organizational concern (Aziri, 2011).

Career satisfaction can be considered the longer-term outcome of having satisfying jobs. Career satisfaction can be expressed by objective indicators like salary, or by people’s subjective experience of being happy with their work over the career span (Ng et al., 2005). In line with Spurk et al. (2011) and Greenhaus et al. (1990), we define it as individuals’ assessment of progress to different career-related goals and successes (e.g., development, income, overall successes). As work is an important aspect in life, which takes a considerable amount of time and energy of people, we argue that life satisfaction is also an important factor. Simply put, people need to earn a living, but additionally, individuals thrive for work that can make and keep them happy, healthy and productive (De Vos and Van der Heijden, 2015).

Life satisfaction concerns the appraisal of one’s life in general and thus refers to one’s global satisfaction with life (Diener et al., 1985; Kjell and Diener, 2021). Since work is such an important aspect in an individual’s life, both job and career satisfaction might relate or spill-over to life satisfaction. Indeed, Beutell and Wittig-Berman (1999) revealed that job and career satisfaction each explained unique variance in life satisfaction. More recently, Hagmaier et al. (2018) confirmed spill-over effects for career satisfaction on life satisfaction, as well as reciprocal effects. Judge et al. (2020) argue for these spill-over effects from job satisfaction to life satisfaction, and reciprocal effects as well. Moreover, as underlined by previous empirical work, all three concepts are known to be affected by workplace factors, such as salary (Beutell and Wittig-Berman, 1999), job design and work conditions (Aziri, 2011) and leadership (see e.g., Belias and Koustelios, 2014; Chang et al., 2020). Therefore, we consider all three concepts as important independent, but also interrelated outcome variables for our study.

### 2.3 Paradoxical leadership and its relationship with job, career and life satisfaction

Several well-known theoretical frameworks applied in the field of leadership (Inceoglu et al., 2018) can explain relationships between paradoxical leadership and individual well-being. First of all, constructive leadership behaviors can, in line with Conservation of Resources (COR) Theory (Hobfoll, 1989), be seen as a “contextual” resource. Such resources may contribute to the accumulation of an employee’s personal resources, such as psychological capital (Caniëls and Stynen, 2022), or job resources like autonomy or job clarity (Fürstenberg et al., 2021). In turn, these are in line with the JDR model expected to relate to favorable outcomes including well-being (Demerouti et al., 2001). As discussed earlier, paradoxical leaders know how to balance control and direction with empowerment and provide their followers leeway (Zhang et al., 2015). Alternatively, one could argue that paradoxical leaders are also strong in investing in the relational bonds with followers, as they can establish closeness with their followers, are capable of attuning to their needs and allow for differentiation in terms of their followers needs (Zhang et al., 2015). Hence, from a social-exchange perspective (Blau, 1964) it could be argued that relational investments made by the paradoxical leader in the follower are reciprocated in job-related attitudes, reflected in job satisfaction. In similar vein it can be argued that paradoxical leaders foster the satisfaction of basic human needs, as put forward in the Self-determination theory (SDT) (Ryan and Deci, 2000) including the need for autonomy (empowerment) and affiliation (relational bonds) (Fürstenberg et al., 2021), which are known to be related to improved well-being in work and beyond (Van den Broeck et al., 2016). Empirically, only one study investigated the relationship of paradoxical leadership with job satisfaction and found a positive relationship (Backhaus et al., 2022). To the authors knowledge, no studies have empirically assessed relationships with career satisfaction and life satisfaction. Obviously, career satisfaction and life satisfaction may in part be contingent upon one’s current job satisfaction as work is an important domain in human life and work-related well-being is related to non-work-related well-being, and both can reciprocally reinforce each other (Bialowolski and Weziak-Bialowolska, 2021). From a theoretical perspective career and life satisfaction can be presumed to be positively affected by paradoxical leadership. Fulfillment of basic needs fosters autonomous motivation, and as people will engage more in activities, either work or non-work-related, that they find interesting and inherently pleasurable (Vansteenkiste et al., 2006; Deci and Ryan, 2008), they will become a more fully functioning person (Vansteenkiste et al., 2020). As this process unfolds it is likely go hand in hand with well-being related outcomes like career and life satisfaction (Walker and Kono, 2018). Given our earlier reasoning, we hypothesize that:

*H1 Paradoxical leadership is positively related with employee job satisfaction (H1a), career satisfaction (H1b) and life satisfaction (H1c).*

### 2.4 Job insecurity as a mediator

Although prior explanations are relevant to understand relationships between paradoxical leadership and employee well-being, they could apply at a broader scale to other constructive leadership styles too, like servant leadership (Caniëls and Stynen, 2022) and may insufficiently account for how this leadership style is apt to address fundamental paradoxical tensions in contemporary workplaces for leaders and their followers. As argued by Zhang et al. (2021) the followers of successful paradoxical leaders may develop an understanding that conflicting demands are inherent to organizational life and may find more productive ways to deal with those uncertainties and ambiguities. To better understand processes in which leaders tune in to workers’ framing of such experiences and shape an appropriate social environment to deal with these accordingly, social-cognitive theories on leadership such as sense-making theory may be valuable (Inceoglu et al., 2018).

Experiences with organizational paradoxes are likely to generate feelings of threat, anxiety and uncertainty resulting in defensive and withdrawal behavior (Lewis, 2000; Smith and Lewis, 2011; Schad et al., 2016; Backhaus et al., 2022). In a general sense, workers may become uncertain regarding one’s role in the organization when exposed to paradoxical tensions (Maitlis and Sonenshein, 2010). Job insecurity, or the perceived threats to the job itself (Greenhalgh and Rosenblatt, 1984) flourishes in a VUCA environment (De Cuyper et al., 2019). Sense-making theory provides a lens to understand how paradoxical leaders can play a role in how followers will experience and understand paradoxical tensions and the associated uncertainties. Specifically, by being a role model to their followers on how to deal with paradoxical tensions and explain these, as a challenge instead of a threat, leaders may enable their followers to cope with uncertainty and ambiguity and hence prevent defensive or negative perceptions or behaviors (Backhaus et al., 2022; Sparr et al., 2022). This line of argumentation may offer a more in-depth explanation for the more general argumentation that paradoxical leadership is a resource that can buffer or diminish the experience of job demands by individuals.

Previous studies have tested the role of paradoxical leadership in buffering job demands, such as role ambiguity (Backhaus et al., 2022). To our knowledge, the effect on uncertainty, like job insecurity, is not studied yet. We expect the line of reasoning to hold for uncertainty factors as well. This leads us to our next hypothesis which suggests a protective role of paradoxical leadership for the job demand of perceived job insecurity:

*H2 Paradoxical leadership is negatively related with job insecurity*

Job insecurity or the perceived uncertainty workers have about the continuity of one’s job (Greenhalgh and Rosenblatt, 1984) is an established work stressor (De Witte, 1999). Specifically, it can be conceptualized as a job demand or the “*physical, psychological, social or organizational aspects of a job that require sustained physical and/or psychological (cognitive or emotional) effort*,” in line with the JD-R model (Bakker and Demerouti, 2007, p. 132). Therefore, job demands have potentially a wear and tear on the human body leading to energy depletion in workers (i.e., exhaustion) (Demerouti et al., 2001). Job hindrances are job demands that are predominantly conceived as solely interfering with people’s work achievement and consuming all energy, yielding no opportunities for psychological growth as job challenges do. Typically, they are seen as threatening and beyond the control of the individual (Cavanaugh et al., 2000; Van den Broeck et al., 2010).

In general, there is consensus that job insecurity, because of its unpredictable and uncontrollable nature, is a hindrance stressor (De Witte et al., 2015; De Witte and Van Hootegem, 2021). Prior research has extensively shown that job insecurity is negatively related to employee well-being over time (De Witte et al., 2016). Meta-analytical studies have found strong negative associations between job insecurity and job satisfaction and life satisfaction (Jiang and Lavaysse, 2018), and empirical studies have also reported negative associations with career satisfaction, as job insecurity is likely to undermine successful career development (Otto et al., 2011; Ngo and Li, 2015). In line we hypothesize:

*H3 Job insecurity is negatively related with employee job satisfaction (H3a), career satisfaction (H3b) and life satisfaction (H3c).*

As a final step for our model, and based on our reasoning thus far, we suggest that job insecurity can act as a mediator in the relationship between paradoxical leadership and employee well-being. Job insecurity can, in line with the JD-R model (Demerouti et al., 2001), be considered a hindrance stressor that impedes employee well-being (De Witte et al., 2015; De Witte and Van Hootegem, 2021). We assume that paradoxical leaders may mitigate the inherent uncertainties in modern workplaces that workers can experience as threatening. As demonstrated earlier, paradoxical leaders can alter workers’ experiences of the working environment, including the experienced resourcefulness (see e.g., Fürstenberg et al., 2021) through stimulating sense-making by either explaining what is at hand or by serving as a role model to their followers (see e.g., Backhaus et al., 2022; Sparr et al., 2022). We argue that these same processes may also alleviate the perceptions of threatening job demands, like job insecurity, are prevented, thereby contributing to employee well-being both in and beyond work. Hence, we more formally hypothesize:

*H4 Job insecurity mediates the relationship between paradoxical leadership and employee job satisfaction (H4a), career satisfaction (H4b) and life satisfaction (H4c).*

**FIGURE 1:**
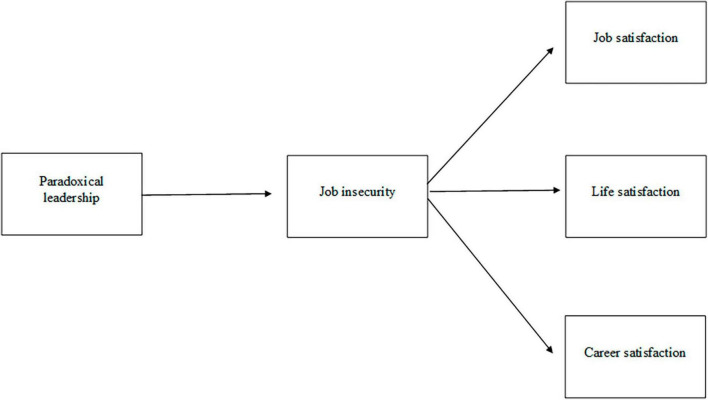
Conceptual model.

The resulting conceptual model for our study is presented in Figure 1.

## 3 Materials and methods

### 3.1 Sample and procedure

To investigate our hypotheses, data were collected by means of an online longitudinal survey. Convenience sampling was applied as respondents were recruited from the professional networks of master students who were subscribed within the broader faculty research line on Sustainable Human Resource Management under supervision of the authors, Prior to data collection approval was granted by the authors’ institutional Ethics Committee (U202101540). As our study was not targeted to specific groups and our master students, who were all employed themselves, had access to different organizations representing substantial variation in sectors and jobs, the aforementioned sampling approach was chosen.

The students contacted Dutch companies and organizations within their professional networks and informed management about the study based on a uniform information letter covering all aspects of the research including the aim and design of the study as well as their rights and how data protection was guaranteed by the researchers. Next, written, formal permission was established from the management of Dutch companies and organizations to participate and recruit respondents within their organization. Organizations determined which departments or teams could participate in the research and informed the selected employees by means of an information letter provided by the researchers, covering all aspects of the research. Eligible respondents were at least 18 years old or older, active as an employee in the organization, and had a company email address. No other inclusion criteria were considered. Eleven organizations granted permission and transferred email addresses of employees to the researchers. Organizations were active in the following sectors: care and welfare (*N* = 3), transportation (*N* = 1), media (*N* = 1), higher and primary education (*N* = 3), industry (*N* = 2), and construction (*N* = 1).

The online survey comprised of a limited set of background variables (e.g., age), organizational and work-related factors (Human Resource practices, paradoxical leadership, job insecurity) and work or career-related outcomes (e.g., job satisfaction, career satisfaction). For all measurements, besides background variables, validated rating scales were used. The survey was developed by the authors and approved by their institutional Ethics Committee. The survey was set up in LimeSurvey, hosted on a server hosted by the authors’ institution. Shortly after selected employees received information from their company, they received an email, which contained a link to the online questionnaire. The email was sent from an email address that was specifically created by the authors and only under their control. Information on the research was again provided online and before respondents could take the survey their informed consent was digitally acquired. The first questionnaire was launched at the end of March 2021 (T0) and reminders were sent after 2 weeks and closed after a month. Of the 3,163 employees that were approached, 1,289 employees responded (40.7%). The follow-up survey was sent 6 months later (T1), to which 741 employees responded (23.4%). In total 287 employees had filled in both surveys and had complete information on all variables included in this study.

The majority of respondents was male (55%), and their average was 47 years. Almost all respondents (92%) were employed in a large organization (250 employees or more). On average organizational tenure of respondents was 15 years. About 74% had obtained either a professional or academic degree in higher education.

### 3.2 Measures

*Paradoxical leadership* was measured at T0 by means of Zhang et al.’s (2015) scale, which comprises of 22 items. An example item was “My supervisor uses a fair approach to treat all subordinates uniformly, but also treats them as individuals” and “Shows a desire to lead, but allows others to share the leadership role” All items were assessed on 5-point Likert scale ranging from 1. “Strongly disagree” to 5. “Strongly agree.” Cronbach’s Alpha was 0.94.

*Job satisfaction* was assessed at T1 by means of a 3-item scale developed by Cammann et al. (1983). An example item was, “Overall, I am satisfied with my current job.” All items were assessed on 7-point Likert scale ranging from 1. “Strongly disagree” to 7. “Strongly agree.” The Cronbach’s Alpha was 0.81.

*Career satisfaction* was measured at T1 using the 5-item Greenhaus et al. (1990) scale. Respondents were asked to indicate their satisfaction with various aspects like career success (e.g., “I am….with the success I have achieved in my career”) on a 5-point Likert scale range from 1. “Very dissatisfied” to 5. “Very satisfied.” Cronbach’s Alpha was 0.82.

*Life satisfaction* was assessed at T1 using Kjell and Diener (2021)’s 3- item scale. Respondents were asked to rate statements about the appraisal of their lives thus far. An example item is “I am satisfied with my life.” A 5-point Likert scale ranging from 1. “Strongly disagree” to 5. “Strongly agree” was used. Cronbach’s Alpha was 0.89.

*Job insecurity* was measured at T0 by means of the job insecurity the subscale developed by Creed et al. (2020) to assess precarious working conditions. An example item was “Are you concerned about losing your current job in the near future?” Respondents could indicate to what extent the described condition applied to them on a 6-point scale ranging from 1. “Not at all” to 6. “A great extent.” Cronbach’s Alpha was 0.78.

*Control variables* that were taken into account are: gender (reference category female), age (in years), organizational tenure (in years), labor market tenure (in years) and educational level. Three levels of educations were distinguished: low (at most secondary school), middle (higher professional education) or high (higher academic bachelor or master or doctorate). The latter category was set as a reference category. In prior research age, gender, tenure and educational level have been identified as demographic predictors of job, career and/or life satisfaction (Martins et al., 2002; Moyes et al., 2006; Park et al., 2010).

### 3.3 Analytical approach

We applied structural equation modeling in two steps (McDonald and Ho, 2002) using Mplus 7.0 (Muthén and Muthén, 2012). First, we conducted Confirmatory Factor Analysis (CFA) to test the measurement model. Next, we applied Path Analysis (PA) to test our hypotheses. Model fit was evaluated by means of the comparative fit index (CFI), the root mean square error of approximation (RMSEA) and the standardized root mean square residuals (SRMR) (Hu and Bentler, 1999). Indications of acceptable model fit are CFI values larger than 0.90 (Bentler, 1990), and RMSEA and SRMR values below 0.08 and 0.10, respectively (Hu and Bentler, 1999).

The hypothesized measurement model comprises of the factors: paradoxical leadership (T0), job satisfaction (T1), career satisfaction (T1), life satisfaction (T1) and job insecurity (T0). The items for job satisfaction, career satisfaction, life satisfaction and job insecurity were loaded on their latent factors, respectively. As the measure of paradoxical leadership comprises of 22 items, the items were parceled (i.e., seven parcels of three to maximum four items) to maintain a favorable ratio between sample size and the number of estimated parameters (Little et al., 2002). The measurement model fitted the data well (χ2(179) = 377.34; *p* < 0.001; CFI = 0.94; RMSEA = 0.06, SRMR = 0.05). To test for common method variance, we conducted Harman’s one-factor (or single-factor) test and compared the fit of that model with our measurement model. The single-factor model resulted in a significantly worse fit (χ2(189) = 1934.24, *p* < 0.001; CFI = 0.46; RMSEA = 0.18 and SRMR = 0.16; Δχ2(10) = 1556.90, *p* < 0.001). As sufficient construct validity was established by our CFA, descriptive results (means, standard deviations and inter-correlations) were computed for all included variables in this study and depicted in Table 1 in the section “4. Results.”

**TABLE 1:** Means, standard deviations, and correlations.

Variable	M	SD	1	2	3	4	5	6	7	8	9	10	11
1. Age	46.98	11.18											
2. Gender	0.56	0.50	−0.24**										
3. Low educational level	0.25	0.44	−0.21**	0.01									
4. Middle educational level	0.46	0.50	−0.10	0.03	−0.54**								
5. High educational level	0.28	0.45	−0.09	−0.03	−0.37**	−0.58**							
6. Organizational tenure	14.81	11.57	0.62**	−0.22**	0.23**	−0.12	−0.09						
7. Labor market tenure	24.82	11.94	0.94**	−0.25**	0.30**	−0.08	−0.20**	0.64**					
8. Paradoxical leadership	3.79	0.60	−0.04	0.06	−0.12*	0.07	0.04	−0.16**	−0.07				
9. Job insecurity	2.21	0.94	0.08	−0.08	−0.06	0.07	−0.02	0.05	0.06	−0.20**			
10. Job satisfaction	5.92	0.97	−0.03	0.04	−0.03	0.06	−0.04	0.03	−0.04	0.30**	−0.28**		
11. Career satisfaction	3.67	0.63	0.19**	−0.14*	−0.09	0.02	0.07	0.13*	0.18**	0.27**	−0.20**	0.47**	
12. Life satisfaction	4.00	0.64	0.02	−0.06	−0.11	0.05	0.06	0.04	−0.01	0.22**	−0.11	0.42**	0.35**

M and SD represent mean and standard deviation, respectively.

*p < 0.05,

**p < 0.01.

Hypotheses were tested by means of PA. In all analyses the confounders were added as covariates. To test hypotheses 1a-c, a first model was estimated in which the three dependent variables were simultaneously regressed on paradoxical leadership. Subsequently, to test hypotheses 2 and H3a-c, a full mediation model was estimated in which job insecurity was added as a mediator to the prior model. Next, to formally examine our mediation hypotheses, as implied by hypotheses 4a-c, the statistical significance of the indirect paths is estimated by means of the MODEL INDIRECT command. To rule out partial mediation, additional direct paths between paradoxical leadership and job satisfaction, career satisfaction and life satisfaction were estimated sequentially.

## 4 Results

### 4.1 Descriptive results

Descriptive results of our study are presented in Table 1.

### 4.2 Hypotheses testing

In a first model, to test hypotheses 1a-c, job satisfaction, career satisfaction and life satisfaction were simultaneously regressed on paradoxical leadership. Support was found for all three hypotheses as paradoxical leadership was positively related to job satisfaction (β = 0.32, *p* < 0.001, 95% CI [0.21, 0.42]), career satisfaction (β = 0.28, *p* < 0.001, 95% CI [0.18, 0.39]) and life satisfaction (β = 0.22, *p* < 0.001, 95% CI [0.11, 0.33]).

Next, a full mediation model was estimated including job insecurity in the role of mediator (χ2(9) = 35.97; *p* < 0.001; CFI = 0.88; RMSEA = 0.10, SRMR = 0.05). Support was provided for hypothesis 2 as paradoxical leadership was negatively related to job insecurity (β = −0.20, *p* = 0.001, 95% CI [−0.31, −0.09]). In addition, job insecurity was negatively related to job satisfaction (β = −0.29, *p* < 0.001, 95% CI [−0.40, −0.18]), career satisfaction (β = −0.23, *p* < 0.001, 95% CI [−0.34, −0.13]) and life satisfaction (β = −0.13, *p* = 0.029, 95% CI [−0.24, −0.01]), corroborating hypotheses 3a-c. This pattern of results suggests mediation as proposed by hypotheses 4a-c. To formally test mediation, indirect effects were computed. The coefficients of the indirect paths between paradoxical leadership via job insecurity to job satisfaction (β = 0.06, *p* = 0.004, 95% CI [0.02, 0.10]) and career satisfaction (β = 0.05, *p* = 0.008, 95% CI [0.01, 0.08]) were both significant, whereas the indirect path to life satisfaction via job insecurity was not significant (β = 0.03, *p* = 0.065, 95% CI [−0.00, 0.05]). Hence, results indicate that job insecurity mediates the associations between paradoxical leadership and job satisfaction and career satisfaction, respectively. As no indication for mediation was found for the association between paradoxical leadership and life satisfaction, hypotheses 4a-b are supported, whereas hypothesis 4c could not be confirmed.

**FIGURE 2:**
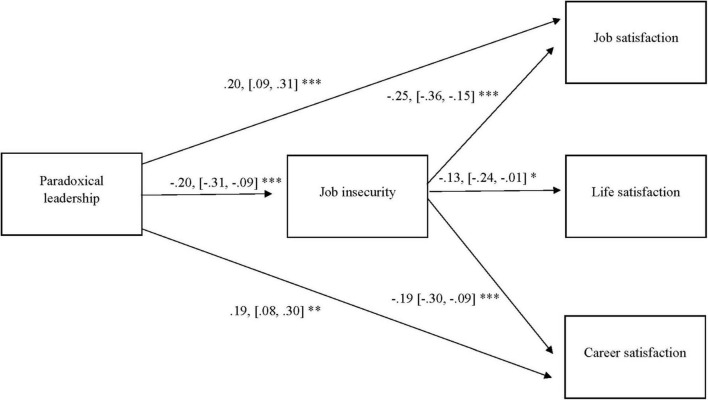
Standardized results path analysis with 95% confidence intervals. *Indicates *p* < 0.05, **indicates *p* < 0.005, ***indicates *p* < 0.001.

To explore the possibility of partial mediation, three additional models were estimated in which sequentially a direct path was added between paradoxical leadership and job satisfaction, career satisfaction and life satisfaction, respectively. In a first model the direct path between paradoxical leadership and job satisfaction was added (χ2(8) = 29.65; *p* < 0.001; CFI = 0.90; RMSEA = 0.10, SRMR = 0.04) and found to be significant (β = 0.13 *p* = 0.013, 95% CI [0.03, 0.23]) and improved model fit (Δχ2(1) = 6.32, *p* < 0.05). Subsequently, a model with a direct path between paradoxical leadership and career satisfaction was estimated (χ2(8) = 30.96; *p* < 0.001; CFI = 0.90; RMSEA = 0.10, SRMR = 0.04). The path was significant (β = 0.12 *p* = 0.026, 95% CI [0.01, 0.22]) and improved model fit (Δχ2(1) = 5.01, *p* < 0.05). Finally, a model with an additional direct path between paradoxical leadership and life satisfaction was estimated (χ2(8) = 34.06; *p* < 0.001; CFI = 0.88; RMSEA = 0.11, SRMR = 0.05). Yet, this path was not significant (β = 0.08 *p* = 0.17, 95% CI [−0.03, 0.19]) and did not improve model fit (Δχ2(1) = 1.91 *p* > 0.05). Hence, it can be concluded that job insecurity partially mediates the association between paradoxical leadership and job satisfaction and career satisfaction, respectively. A model in which both direct paths are simultaneously added, fitted the data well (χ2(7) = 17.97; *p* < 0.001; CFI = 0.95; RMSEA = 0.07, SRMR = 0.03). The standardized coefficients of this final model are depicted in Figure 2. The R-square for the dependent variables career satisfaction, job satisfaction, life satisfaction and job insecurity are: 0.16, 0.13, 0.04, and 0.04, respectively. Concerning the control variables only the low educational level was significantly related to career satisfaction (β = −0.17, *p* = 0.018, 95% CI [−0.31, −0.03]). All other control variables were unrelated to any of these dependent variables.

The standardized results of our path analysis are summarized in Figure 2 below.

## 5 Discussion

Developments in society and at work, such as the increased use of artificial intelligence, and flexibilization of labor, but also broader economic uncertainties are challenging organizations and their employees (Yam et al., 2023). These developments are inherently related to tensions that are paradoxical in nature, and put increasing and new demands on all personnel layers in organizations with the potential to undermine individual well-being (Zhang et al., 2021; Nilsen and Kongsvik, 2023). Due to the diverging interests of the different stakeholders involved (such as client or customer, worker, higher management, legislation, and environmental interests), new and different behaviors are required in the management of organizations to continue their business and deal with all these emerging paradoxical tensions (Zhang et al., 2022).

Leaders are crucial for employee well-being (Das and Pattanayak, 2023) and play an important role in dealing with these tensions, not only for themselves, but also as a role model for their followers in addressing these. Paradoxical leadership (Zhang et al., 2015), a new concept within the leadership literature, has the potential to make a difference in these turbulent times. Knowledge on the impact of paradoxical leadership, for example on worker well-being, is however still scarce. In this study, we contributed to that emerging stream in the literature by studying the role of paradoxical leadership on worker well-being in terms of job, career and life satisfaction and investigating to what extent these linkages can be explained by the mitigation of workers’ experienced job insecurity. To explain the proposed mechanisms, we relied on the JD-R framework (Demerouti et al., 2001) as well as sense-making theory (Weick, 1995). We investigated these relationships by administrating an online survey among Dutch workers from eleven different profit and non-profit organizations. We tested the model with structural equation modeling using a time-lagged design.

### 5.1 Theoretical implications

First of all, our results indicated that paradoxical leadership positively affects all three outcomes directly (job, career, and life satisfaction) herewith supporting hypotheses 1a, b and c. These findings corroborate our propositions, suggesting that paradoxical leadership can be seen as a contextual resource that triggers the accumulation of other personal resources in line with COR-theory (Hobfoll, 1989), either because it fulfills peoples’ basic needs as SDT proposes, or because it stimulates vigor in workers as the JD-R model implies. Under such momentary conditions people can thrive and it is understandable that well-being, as indicated by job satisfaction, is able to flourish. Yet, findings also suggest that career satisfaction and life satisfaction, which imply a longer time window, are fostered. Probably, these can be explained in line with SDT, which suggests that basic needs satisfaction is a leverage for making more autonomous choices in one’s career and life as a whole. As a consequence, subjective assessments of satisfaction with one’s career and life are experienced as more favorable. These findings support the results of Backhaus et al. (2022) regarding the positive effect of paradoxical leadership on job satisfaction. Moreover, we add to the literature on paradoxical leadership and its consequences for worker well-being as we found positive effects on career and life satisfaction as well.

Paradoxical leadership appeared to be negatively associated with job insecurity, which supports hypothesis 2. This is in line with our reasoning based on sense-making theory that paradoxical leadership can help workers to be able to deal better with job demands in terms of challenges, or uncertainty. Specifically, and building on the relevant notions of sense-making theory (Zhang et al., 2021), we argued that paradoxical leadership can help followers to deal better with demands in such a way that they aid in framing stressors as challenges which remain more under workers’ control. By making sense of uncertainties and putting them in perspective, such uncertainties are less likely to trigger hindrance appraisals of stressors like perceived job insecurity, which are experienced as out of workers’ control. This finding also contributes to the literature on the work-related antecedents of job insecurity, which has received less attention compared the consequences of job insecurity (Shoss, 2017). In addition, it adds to sense-making theory as a relevant theoretical framework to aid our understanding regarding the role of shared work-related factors at the meso-level, like leadership, in explaining employee perceptions of the psycho-social working environment, like job insecurity. However, currently we can only implicitly confirm this line of argumentation. To test these propositions more explicitly, future research could include the measurement of sense-making processes in the empirical model. Measuring sense-making directly can be difficult, and quite paradoxical in itself, requiring conscious choices of what and how it is measured (Allard-Poesi, 2005). Previous empirical research made use of a combination of tailor-made survey data and open-ended questions (Bartunek et al., 2006). Alternatively, qualitative research may also aid in better understanding how such process develops.

Next, job insecurity was directly and negatively related to all three outcomes (job, career and life satisfaction) as well, thereby supporting hypothesis 3a, b and c. These results align with the argument based on the JD-R model (Demerouti et al., 2001) that job insecurity can be considered a job demand that diminishes positive outcomes, because of the energy depletion process that has been triggered. Such a demand is consuming available energy resources of the individual without yielding sufficient opportunities to replenish these resources again in time (Bakker and Demerouti, 2007). Not only is well-being in one’s current job context affected in a negative way, but it can apparently spill over to satisfaction appraisals of one’s entire career and life in general as well. Job insecurity has previously been identified as an important hindrance stressor with negative consequences on several aspects for individuals and organizations alike (Sverke et al., 2002; Jiang and Lavaysse, 2018, Langerak et al., 2022), which is again confirmed by our results. In particular the implication for career satisfaction broadens the existing evidence base, as this outcome has barely been studied in relation to job insecurity before.

Finally, job insecurity mediates the effect of paradoxical leadership on job and career satisfaction, but not on life satisfaction, herewith supporting hypotheses 4a and b, but not hypothesis 4c. This means that our presumed pathway through the mitigation of job insecurity holds for work related outcomes, but does not account for the broader concept of life-satisfaction. There are two plausible explanations. First of all, life satisfaction is affected by many different non-work-related factors (Near et al., 1984), and varies across cultures (Oishi et al., 2009). In this respect, life events like sickness, personal setbacks in relationships, but also the current larger societal circumstances (e.g., war, economic recession, the COVID crisis) might have an unknown impact. Also job insecurity has been empirically shown to be a more sizeable predictor of job satisfaction than life satisfaction. Second, it is possible that job and career satisfaction may act as a mediating mechanism between work-related factors like leadership, job demands and life satisfaction. As also raised mentioned earlier, spill-over effects between these types of satisfaction have been detected (Hagmaier et al., 2018; Judge et al., 2020) but not explicitly tested in our research. Alternatively, the positive effect of paradoxical leadership on life satisfaction might be explained by different mechanisms. COR-theory suggests that paradoxical leadership is a contextual resource that may trigger personal resource accumulation, for example, in terms of one’s psychological capital (PsyCap; Luthans et al., 2007). Enhanced optimism, hope and resilience could contribute to more life satisfaction. SDT would emphasize the role of basic need fulfillment and the making of autonomous choices in life could be a plausible alternative. This interpretation suggests that resource-oriented explanations may be more fruitful to explain life satisfaction compared to work-related well-being outcomes, like job and career satisfaction. In addition, it should also be noted that regarding career and job satisfaction, we only established indications for partial mediation. This suggests that also other processes (e.g., resource-oriented explanations), or other job demands (e.g., role conflict, role ambiguity) may be useful alternatives to consider in future research. Nevertheless, our study is one of the few that investigates to what extent sense-making processes involving the malleability of perceived job demands may act as a mediating mechanism. Whereas Fürstenberg et al. (2021) found support for the mediating role of job resources, our study suggests that paradoxical leaders may also shape the perceptions of job demands in their followers. Therefore, efforts to further the alignment of sense-making theory with theories on the psychosocial working environment like the JDR-model might help to better understand how contextual factors, like leadership, may shape perceptions of the demanding aspects of work over time.

### 5.2 Practical implications

The results of our study also have implications for practice, as paradoxical leadership seems to play a positive role for worker well-being in these turbulent times. It is therefore recommended that organizations get acquainted with this rather new leadership behavior and let their leaders learn how to develop it. This leadership behavior can help to directly enhance worker well-being in terms of job, career and life satisfaction, and could also help people from reframing uncertainties in the workplace in a way that prevents hindrance appraisals of job insecurity. Although job insecurity is difficult to control, paradoxical leadership can be trained, which can be facilitated by the organization. Moreover, worker well-being can be incorporated in the performance appraisals for leaders to emphasize its importance and to follow up with explicit developmental goals for the desirable leadership behavior. Worker well-being, such as job, career and life satisfaction, is not only important for its own good, but also known to be an important prerequisite for good organizational functioning (e.g., resilience, innovation and viability) (see, e.g., Harter et al., 2003; Kuntz et al., 2016; Ungar, 2021). It therefore deserves explicit attention.

### 5.3 Limitations and avenues for future research

The current study has its limitations as well. First, although our model is time-lagged, a longitudinal design with three measurement waves could test the mediating role of job insecurity more rigorously, in particular how job insecurity levels may change over time. Related, many contextual factors (e.g., regional unemployment rate, financial turmoil in companies) can influence perceived job insecurity (Shoss, 2017), which we did not account for, and therefore endogeneity cannot be ruled out. Future research can consider taking alternative, contextual variables as confounders into account. Second, our measures consisted of self-reports among employees, which enhances the risk for mono-method bias (Donaldson and Grant-Vallone, 2002). In our design, this risk has been mitigated by guaranteeing anonymity for respondents, herewith creating a context for honest responses as much as possible, and giving full autonomy to withdraw from the study at any time (Podsakoff et al., 2012). In addition, we used survey scales with varying scale anchors which can prevent stylistic answering. However, future research could make use of multisource reports. Ratings of the leaders or supervisors of these employees could be valuable as well, also in terms of the discrepancies between these ratings when it comes to the relationship with relevant outcomes concerning worker well-being (see e.g., Kopperud et al., 2014; Mazzetti et al., 2016). Alternatively, one could also aim to investigate these processes in the context of teams and investigate these relationships from a multi-level perspective. Finally, in this study we opted for a time lag of 6 months. Potentially, as participants lost connection with the study over this time interval, this resulted in increased drop-out and hence a low response on our follow-up measurement. Generalization of findings should be done with caution.

Knowledge on the influence of paradoxical leadership is only in its infancy, but seems promising based on our results. For future research alternative theoretical perspectives can be further explored including SDT, but also social-exchange related frameworks and related concepts like leader-membership exchange (LMX) (Dansereau et al., 1975; Graen and Uhl-Bien, 1995) to better understand the underlying mechanisms of the positive effects. In line with this, the alignment of Sense- making theory (Weick, 1995) with other frameworks is relevant to explore too. Relevant contributions can also be sought in studying other outcomes that are of interest to the organizations, such as their innovative capacity (Camisón and Villar-López, 2014), but also longer-term viability (Stjernberg and Philips, 1993).

Finally, the value of this leadership behavior when compared to other contemporary leadership behaviors could be explored. In particular to styles that often imply diverging behaviors from leaders, such as ambidextrous leadership (Probst et al., 2011; Rosing et al., 2011), and engaging leadership (Schaufeli, 2015) versus disengaging leadership (Nikolova et al., 2021).

## Data availability statement

The datasets presented in this article are not readily available because respondents have not consented to share the data with third parties. Requests to access the datasets should be directed to DS, dave.stynen@ou.nl.

## Ethics statement

The studies involving human participants were reviewed and approved by the Research Ethics Committee (cETO) of the Open Universiteit (Netherlands). The patients/participants provided their written informed consent to participate in this study.

## Author contributions

Both authors listed have made a substantial, direct, and intellectual contribution to the work, and approved it for publication.
